# Are national treatment guidelines for falciparum malaria in line with WHO recommendations and is antimalarial resistance taken into consideration? – A review of guidelines in non‐endemic countries

**DOI:** 10.1111/tmi.13715

**Published:** 2022-01-13

**Authors:** Marc T. Visser, Rens Zonneveld, Thomas J. Peto, Michele van Vugt, Arjen M. Dondorp, Rob W. van der Pluijm

**Affiliations:** ^1^ Amsterdam University Medical Centers University of Amsterdam Amsterdam The Netherlands; ^2^ Department of Medical Microbiology and Infection Prevention Amsterdam University Medical Centers University of Amsterdam Amsterdam The Netherlands; ^3^ Mahidol‐Oxford Tropical Medicine Research Unit Faculty of Tropical Medicine Mahidol University Bangkok Thailand; ^4^ Centre for Tropical Medicine and Global Health Nuffield Department of Medicine University of Oxford Oxford UK; ^5^ Department of Internal Medicine & Infectious Diseases Amsterdam University Medical Centers University of Amsterdam Amsterdam The Netherlands

**Keywords:** guidelines, malaria, review, treatment, WHO

## Abstract

**Objective:**

*Plasmodium falciparum* infections are a relatively rare but potentially deadly disease found in returning travellers. We compare the national treatment guidelines of non‐endemic countries with the WHO guidelines for the treatment of *Plasmodium falciparum* infections.

**Methods:**

Review. We identified non‐endemic countries with an incidence rate of imported malaria of at least one per 100,000 population and at least 50 cases annually. Using PubMed and Google Search, we reviewed national guidelines published before 1 March 2021.

**Results:**

Thirteen guidelines were identified. For uncomplicated falciparum malaria, 11 of 13 countries (85%) recommend an artemisinin‐based combination therapy as first‐line regimen in adults, of which artemether–lumefantrine was the most common. For severe malaria, all guidelines recommend the use of intravenous artesunate. Only three countries adjust treatment recommendations based on expected artemisinin resistance.

**Conclusion:**

Treatment guidelines for uncomplicated falciparum malaria in non‐endemic countries generally adhere to WHO recommendations but often fail to mention the risk of drug resistance in returning travellers. Artemisinin‐based Combination Therapies (ACTs) should be the first choice for all uncomplicated malaria cases. Furthermore, the choice between ACTs should be based on regional resistance patterns.

## INTRODUCTION

Malaria remains an important cause of morbidity and mortality in large parts of the world. WHO currently recommends the use of artemisinin‐based combination therapies (ACTs) for all *Plasmodium* species. These ACTs are artemether–lumefantrine, artesunate–amodiaquine, artesunate–mefloquine, artesunate–sulphadoxine–pyrimethamine, dihydroartemisinin–piperaquine and artesunate–pyronaridine [1]. These ACTs are recommended as both first‐ and second‐line treatment options, because alternative antimalarial drugs such as chloroquine, quinine and doxycycline are less effective and have more adverse effects [2]. In addition, quinine‐based regimens are given for 7 days, limiting treatment adherence.

*Plasmodium falciparum* infections are a relatively rare but potentially fatal cause of disease found in returning travellers. Even though Europe is a non‐endemic region, each year around 7300 travellers return with malaria [3]. Different countries write different guidelines, in part based on the local availability of some antimalarials. Physicians might follow these guidelines instead of the recommendations made by the WHO. If these guidelines are not in line with WHO recommendations, patients might receive suboptimal malaria treatment. This could increase the risk of treatment failure, in which the patient will suffer from a recrudescent episode of malaria. In addition, suboptimal treatment could result in progression of the uncomplicated malaria infection into a severe malaria infection. Complicated or severe malaria itself should be treated with parenteral treatments instead of oral antimalarials [1]. In the case of severe malaria, intravenously administered artesunate should be chosen over quinine, if available [4, 5].

Antimalarial resistance should be taken into account when choosing an antimalarial treatment. For instance, chloroquine‐resistant *Plasmodium falciparum* is already widely established in most parts of the world [6]. In addition, artemisinin and partner drug resistance are spreading in the Greater Mekong Subregion (GMS), leading to treatment failures of ACTs such as dihydroartemisinin–piperaquine and artesunate–mefloquine [7, 8, 9]. Considering the high treatment failure rates in the GMS, travellers returning from this region should be treated taking into account the prevalence of resistance or at least be monitored more intensely as the likelihood of treatment failure is higher. According to the WHO guidelines, an alternative regimen should be considered as a second‐line treatment in case of treatment failure [1]. In the case of a returning traveller living in a non‐endemic country, a recurrent infection occurring in a non‐endemic country is by definition a treatment failure.

Of the five malaria species known to cause infections in humans, *Plasmodium falciparum* is associated with the highest morbidity and mortality. The aim of this review is therefore to report how closely national treatment guidelines of non‐endemic countries follow WHO recommendations for *Plasmodium falciparum* malaria treatment, taking into consideration disease severity and artemisinin resistance (Table 1).

**TABLE 1:** World Health Organization treatment recommendations for *P*. *falciparum* malaria

	Groups			
	Adults	Children	Pregnant women	
			1^st^ trimester	2^nd^/3^rd^ trimester
Uncomplicated				
1^st^	ACT^a^	ACT^a^	Quinine + clindamycin	ACT^a^
2^nd^			Artesunate + clindamycin	
Severe (parenteral administration)				
1^st^	Artesunate	Artesunate	Artesunate	Artesunate
2^nd^	Artemether	Artemether	Artemether	Artemether
3^rd^	Quinine	Quinine	Quinine	Quinine

Note

Treatment of severe *P*. *falciparum* malaria should be parenteral for at least 24 h and until the patient can tolerate oral medication. Treatment should be completed with an ACT for 3 days, as is the standard treatment for uncomplicated *P*. *falciparum* malaria.

Abbreviation: ACT, artemisinin‐based combination therapy.

^a^ ACTs recommended by the WHO include: artemether + lumefantrine, artesunate + amodiaquine, artesunate + mefloquine, dihydroartemisinin + piperaquine, artesunate + sulphadoxine–pyrimethamine, artesunate + pyronaridine.

## METHODS

We identified non‐endemic countries with an incidence rate of imported malaria of at least 1 per 100,000 population and at least 50 cases annually, based on epidemiological data from the European Centre for Disease Prevention and Control and countries respective health organisations [3, 10, 11, 12, 13]. Additionally, the United States of America were included as the guidelines from their Centre for Disease Control might have been regarded as a reference for some countries without guidelines of their own.

National malaria guidelines were identified using Google Search and PubMed using terms such as ‘Malaria’ and ‘Guideline(s)’ and the name of the country of interest. We included the latest guidelines published before 1 March 2021. Where needed, Google Translate was used.

Guidelines on prophylaxis were excluded, as were sub‐national guidelines. We reviewed the guidelines while focussing on first‐ and second‐line treatments for uncomplicated and severe or complicated *Plasmodium falciparum* malaria in adults, children and pregnant women. We also assessed whether the prevalence of antimalarial resistance in the country of transmission was considered when choosing an antimalarial. Definitions of (un)complicated malaria and artemisinin drug resistance can be found in the WHO guidelines on malaria treatment [1]. Additionally, if artemether–lumefantrine was recommended, we assessed whether the recommendation was made to take this with a fatty drink or food. Finally, we verified if follow‐on treatment for severe malaria was proposed in the form of ACTs.

## RESULTS

Based on the incidence of imported malaria, as specified in the Methods section, we selected 17 countries, of which 13 were found to have a national guideline: Australia [14], Belgium [15], Canada [16], Denmark [17], France [18, 19, 20], Germany [21], Ireland [22], Italy [23], The Netherlands [24], Spain [25], Switzerland [26], the United States of America [27] and the United Kingdom [28]. Two countries (Belgium and Italy) recommend the use of an ACT and refer to the WHO guideline for further details. We did not find a national guideline for Portugal, and for Sweden, only sub‐national guidelines were found.

### Uncomplicated malaria

For uncomplicated *Plasmodium falciparum* malaria [Table 2] in adults, 11 of 13 countries recommend an ACT as first‐line treatment, predominantly artemether–lumefantrine (nine countries), followed by dihydroartemisinin–piperaquine (four countries). Three countries include atovaquone–proguanil as first‐line treatment. Canada recommends quinine combined with doxycycline as first‐line treatment. The United States of America advise (hydro)chloroquine for infections acquired in areas with chloroquine‐sensitive *Plasmodium falciparum*. An ACT is the first choice for chloroquine‐resistant *Plasmodium falciparum*. Not a single country advises more than two ACTs as potential first‐line treatments.

**TABLE 2:** Treatment options for uncomplicated *P*. *falciparum* malaria

Country	Groups				AL combined with food?	Annual malaria cases (per 100,000)
	Adults	Children	Pregnant women			
			1^st^ trimester	2^nd^/3^rd^ trimester		
**Europe**						
Belgium						
1^st^	ACT	*Conform WHO*	*Conform WHO*	*Conform WHO*	Yes	357 (3.1)^g^
Denmark						
1^st^	AL	AL	Q + dox/clin	AL	Yes	64 (1.1)^g^
2^nd^	DHA‐PPQ	DHA‐PPQ		DHA‐PPQ		
3^rd^	AQ‐PG	AQ‐PG		AQ‐PG		
4^th^	Q + dox/clin	Q + dox/clin		Q + clin		
France						
1^st^	AL, DHA‐PPQ	AL, DHA‐PPQ	AQ‐PG	AL, DHA‐PPQ	Yes	2840 (4.2)^g^
2^nd^	AQ‐PG	AQ‐PG, MQ	Q			
3^rd^		Q	*(AL)* ^a^			
Germany						
1^st^	AL, AQ‐PG, DHA‐PPQ^b^	AL, AQ‐PG	Q + clin	AL	Yes	896 (1.1)^g^
Ireland						
1^st^	AL	–	Q + clin, A + clin	AL, AMQ, Q + clin, A + clin	No	60 (1.2)^g^
2^nd^	AMQ					
3^rd^	Q + dox/clin					
4^th^	AQ‐PG					
Itay						
1^st^	ACT	*Conform WHO*	*Conform WHO*	*Conform WHO*	n/a	722 (1.1)^g^
The Netherlands						
1^st^	AL	AL	AL	AL	Yes	252 (1.5)^g^
2^nd^	AQ‐PG	AQ‐PG				
3^rd^	MQ	MQ				
4^th^	Q + clin/dox					
Portugal						
1^st^	No data				Yes	102 (1.0)^g^
Spain						
1^st^	AL, AQ‐PG, DHA‐PPQ	AL, AQ‐PG	Q + clin	AL, Q + clin	Yes	851 (1.8)^g^
2^nd^	Q + dox	Q + clin				
3^rd^						
Sweden						
1^st^	No data				n/a	189 (1.9)^g^
Switzerland						
1^st^	AL, DHA‐PPQ^c^	AL, DHA‐PPQ^c^	Q + clin	AL^c^	No	435 (5.2)^h^
2^nd^	AQ‐PG, MQ	AQ‐PG, MQ		Q + clin, MQ		
The United Kingdom						
1^st^	AL	AL, DHA‐PPQ	Q + clin	AL	Yes	1656 (2.5)^g^
2^nd^	DHA‐PPQ	Q + dox	AQ‐PG			
3^rd^	Q + dox, AQ‐PG					
**North America**						
Canada^d^						
1^st^	AQ‐PG, Q + dox	–	–	–	n/a	488 (1.4)^i^
2^nd^	Q iv					
3^rd^	A iv					
The United States of America						
1^st^	(H)CQ^e^	(H)CQ^e^	(H)CQ^e^	(H)CQ^e^	No	2161 (0.7)^j^
2^nd^	AL	AL	Q + clin	AL		
3^rd^	AQ‐PG, Q + tetra/dox/clin	AQ‐PG, Q + tetra/dox/clin	MQ	Q + clin		
4^th^	MQ	MQ	AL	MQ		
**Oceania**						
Australia						
1^st^	AL^f^	AL^f^			Yes	Approximately 500 (2.1)^k^
2^nd^	AQ‐PG	AQ‐PG				
3^rd^	Q + dox	Q + dox/clin	Q + clin	Q + clin		

Abbreviations: (H)CQ, (hydro)chloroquine; A, artesunate; ACT, artemisinin‐based combination therapy; AL, artemether + lumefantrine; AMQ, artesunate + mefloquine; AQ‐PG, atovaquone–proguanil; clin, clindamycin; DHA‐PPQ, dihydroartemisinin + piperaquine; dox, doxycycline; iv, intravenous; MQ, mefloquine; n/a, not applicable; Q, quinine; tetra, tetracyclin; WHO, World Health Organization.

^a^ May be considered.

^b^ If >2% parasitemia: AL or DHA‐PPQ preferred over AQ‐PG.

^c^ Due to resistance related treatment failures from the Greater Mekong Subregion, AQ‐PG is preferred over AL or DHA‐PPQ if infection was acquired in this region

^d^ Artemisinin‐based combination therapies not yet available in Canada. When they do they will become first‐line treatment.

^e^ For chloroquine‐sensitive *P. falciparum*, use (H)CQ. If not, use to alternative treatment options.

^f^ Seek expert advice for patients with malaria caused by *P. falciparum* (either alone or with other species) acquired from the Greater Mekong Subregion who respond slowly to AL. Options include prolonging treatment or switching to second and third line treatments.

^g^ European Centre for Disease Prevention and Control; 2018.

^h^ Eperon G, et al. Malaria cases in Switzerland from 2005 to 2015 and recent rise of imported Plasmodium vivax malaria; 2017.

^i^ Canada Malaria Network; 2016.

^j^ Centers for Disease Control and Prevention; 2017.

^k^ New South Wales Health; 2016.

Eleven countries specify treatment recommendations for children. In seven of those guidelines, artemether–lumefantrine is the first choice, followed by dihydroartemisinin–piperaquine (three countries) and atovaquone–proguanil (two countries).

Treatment protocols for pregnant women are specified in 12 guidelines. Only The Netherlands recommend the use of artemether–lumefantrine as first‐line treatment in the first trimester. Quinine is advised six times as first‐line treatment in the first trimester, (hydro)chloroquine and atovaquone–proguanil both once. In the second or third trimester, 10 countries recommend the use of an ACT as first‐line treatment, most often artemether–lumefantrine.

Nine of the 12 countries that recommend artemether–lumefantrine as an option, recommended to take it with food or a fatty drink.

### Severe malaria

Regarding severe *Plasmodium falciparum* malaria [Table 3], all countries advise intravenous artesunate as first‐line treatment. In the nine guidelines where treatment options for pregnant women are specified, only Spain advises intravenous quinine as first‐line treatment in the first trimester. All nine countries advise intravenous artesunate as first‐line treatment for infections during the second or third trimester of pregnancy. The 11 countries that specify treatment for severe malaria in children all advise intravenous artesunate as first‐line treatment. Four of 11 countries combine quinine, be it as first or second choice for severe malaria, with antibiotics such as doxycycline or clindamycin. All countries recommend oral therapy as follow‐on medication.

**TABLE 3:** Treatment options for severe *P*. *falciparum* malaria

Country	Groups				Follow‐on treatment
	Adults	Children	Pregnant women		
			1^st^ trimester	2^nd^/3^rd^ trimester	
**Europe**					
Belgium					
1^st^	*Conform WHO recommendations*				
Denmark					
1^st^	A iv	**–**	A iv	A iv	Oral therapy
2^nd^	Q iv + clin		Q iv + clin	Q iv + clin	
France					
1^st^	A iv^a^	A iv^a^	A iv^a^	A iv^a^	Oral therapy
2^nd^	Q iv	Q iv	Q iv	Q iv	
Germany					
1^st^	A iv	A iv	A iv	A iv	AQ‐PG
2^nd^	Q iv + dox/clin	Q iv + clin			
Ireland					
1^st^	A iv	**–**	**–**	**–**	Oral A or Q + dox
2^nd^	Q iv + dox/clin				
Italy					
1^st^	*Conform WHO recommendations*				
The Netherlands					
1^st^	A iv	A iv	A iv	A iv	Oral therapy
Portugal					
	No data				
Spain					
1^st^	A iv	A iv	Q iv + clin	A iv	Oral therapy (AL for pregnant women)
2^nd^	Q iv + dox	Q iv + clin		Q iv + clin	
Sweden					
	No data				
Switzerland					
1^st^	A iv	A iv	A iv	A iv	AL, DHA‐PPQ, AQ‐PG^b^
2^nd^	Q iv	Q iv	Q iv	Q iv	
The United Kingdom					
1^st^	A iv	A iv + broad spectrum AB	**–**	**–**	Oral therapy
2^nd^	Q iv	Q iv + broad spectrum AB			
**North America**					
Canada					
1^st^	A iv	A iv	**–**	**–**	Full dose of oral AQ‐PG or Q + dox/clin
2^nd^	Q iv	Q iv			
The United States of America					
1^st^	A iv	A iv	**–**	**–**	Oral therapy (AL 1st choice)
**Oceania**					
Australia					
1^st^	A iv^c^	A iv^c^	A iv^c^	A iv^c^	Oral therapy
2^nd^	Q iv	Q iv	Q iv	Q iv	

Abbreviations: A, artesunate; AB, antibiotics; AL, artemether + lumefantrine; AQ‐PG, atovaquone–proguanil; clin, clindamycin; DHA‐PPQ, dihydroartemisinin + piperaquine; dox, doxycycline; im, intramuscular; iv, intravenous; MQ, mefloquine; Q, quinine.

^a^ Intravenous quinine is the first‐line treatment for travellers from Southeast Asia, when combined with artemisinin.

^b^ If the infection has been acquired in Southeast Asia, AQ‐PG might be the preferred sequential agent.

^c^ Seek expert advice for patients with severe *P*. *falciparum* malaria acquired in the Greater Mekong Subregion. Combination therapy with intravenous artesunate plus intravenous quinine is now recommended for these patients. Do not delay therapy if only one of the two intravenous drugs is immediately available.

### Resistance

Only three guidelines (Australia, France and Switzerland) incorporate artemisinin resistance patterns in the GMS into their recommendations on treating patients from this region. The French guideline advises the combination of intravenous quinine and artesunate instead of artesunate monotherapy for severe malaria in this group. The Australian guideline also recommends to take the origin of the infection into account when treating a case of severe malaria and recommended seeking expert advice. Additionally, the Australian guideline offers the options of prolonging treatment with artemether–lumefantrine, switching to atovaquone‐proguanil or switching to quinine combined with doxycycline or clindamycin for uncomplicated *Plasmodium falciparum* malaria in travellers from the GMS. The Swiss guideline suggests taking atovaquone–proguanil over artemether–lumefantrine or dihydroartemisinin–piperaquine for uncomplicated, or as follow‐on medication for severe *Plasmodium falciparum* malaria due to resistance‐related treatment failures in the GMS. The other countries do not adjust their recommendations for travellers from a region with artemisinin resistance.

## INTERPRETATION

Malaria remains a potentially deadly disease in returning travellers. Drug resistance patterns and treatment options are rapidly evolving, stressing the need for up‐to‐date guidelines on malaria treatment. This study provides an overview of the state of malaria treatment guidelines across a multitude of non‐endemic counties. Comparing national treatment guidelines with WHO recommendations showed many countries still recommending suboptimal treatment regimens. Improvements can be made by prescribing ACTs as first‐line treatment and by taking the origin of infection into account.

### Uncomplicated malaria

ACTs are already generally accepted as the best treatment regimens for uncomplicated *Plasmodium falciparum* malaria. They are more effective and have fewer adverse effects than other antimalarials such as atovaquone–proguanil, quinine or mefloquine [29, 30, 31, 32]. The WHO recommends a total of six ACTs, of which countries can select first‐ and second‐line treatment [1]. While most countries included in this review advise the use of an ACT as first‐line treatment, four of 13 countries still advise non‐ACTs as (alternative) first‐line treatment.

Canada still recommends the use of quinine in combination with doxycycline or clindamycin as first‐line treatment, due to unavailability of ACTs. This combination of drugs is accountable for more adverse effects than ACTs [2]. Atovaquone–proguanil has fewer adverse effects than quinine but is also less effective than ACTs and should therefore not be a first‐line treatment [33, 34]. Three of four countries recommending mefloquine as one of the treatment options do so as a monotherapy, while its efficacy is significantly better when combined with artesunate [1, 35]. The United States of America differentiates between chloroquine‐sensitive and ‐resistant forms of *Plasmodium falciparum*, as chloroquine‐sensitive *Plasmodium falciparum* is still endemic in parts of Latin America. Their guideline suggests different treatments for those groups, preferring (hydroxy)chloroquine as first‐line treatment when possible. However, WHO recommendations make no distinction between chloroquine‐resistant strains of *Plasmodium falciparum* and recommend using an ACT for all forms of *Plasmodium falciparum* malaria [1].

Guidelines specifying treatment protocols for children did not differ much from adult treatment protocols. WHO recommends the use of ACTs here as well but with different dosages depending on body weight [1]. In 10 countries, an ACT is the first‐line treatment for uncomplicated malaria in children, showing treatment protocols for children are, at least when specified, often in line with WHO recommendations.

The use of ACTs in the first trimester of pregnancy is still debated. Current WHO recommendations still advise the use of quinine with clindamycin in the first trimester due to limited clinical safety data for ACTs [1]. An ACT may be given as an alternative, as is oral artesunate with clindamycin. Currently, most countries adhere to this recommendation. During the second and third trimesters, almost all countries advise an ACT as first‐line treatment, conforming to WHO recommendations.

In general, artemether–lumefantrine was the most used ACT. Higher levels of lumefantrine are associated with lower rates of treatment failure [36]. For this reason, the WHO guidelines recommend artemether–lumefantrine to be taken directly after food or with a fatty drink such as milk, which is also addressed in most guidelines.

### Severe malaria

Regarding severe malaria, all countries included in this review recommend intravenous artesunate over intravenous quinine. Parenteral artesunate is superior to quinine in both adults and children [4, 5]. Guidelines specifying treatment protocols for children and pregnant women also advise intravenous artesunate for those groups. Only in Spain is intravenous quinine the preferred regimen during the first trimester.

Severe malaria should be treated with oral follow‐on medication to eradicate the remaining parasitaemia. WHO recommends a full dose of effective ACTs orally after the initial parenteral treatment. Combining the data from uncomplicated malaria treatment protocols, we found 10 of 13 countries including an ACT as first‐line follow‐up treatment. Three countries explicitly advise a non‐ACT as follow‐on medication: atovaquone–proguanil (Germany), atovaquone–proguanil or quinine with doxycycline/clindamycin (Canada), and artesunate or quinine with doxycycline (Ireland). Still, as described above, an ACT is superior to non‐ACTs such as atovaquone–proguanil or quinine as follow‐on medication.

### Resistance

With artemisinin and partner drug resistance spreading in the GMS, travellers returning from this region could be infected by an artemisinin and partner drug‐resistant *Plasmodium falciparum* malaria strain. In a recent prospective study, conventional treatment with dihydroartemisinin–piperaquine showed therapy failure rates up to 93% in the GMS [7]. Only three of the guidelines found differentiate between travellers returning from the GMS and other parts of the world. Two of those suggest combining intravenous artesunate and quinine for severe malaria, a combination proven to be safe [37]. However, randomised evidence of its benefit above treatment with artesunate alone in artemisinin‐resistant severe malaria is currently still lacking.

So far, artemisinin resistance seems to have been contained to the GMS. And although some partner drugs have been used in monotherapy at large scale in the last decades, this has not led to widescale ACT failure outside of the GMS [8]. However, a recent report on the independent emergence of artemisinin resistance in Rwanda is of great concern [38, 39]. In the case of (partial) artemisinin resistance, a larger proportion of parasites survive the initial 3 days of treatment. This in turn facilitates the selection of partner drug resistance.

Again, we believe taking a travellers' origin into consideration is necessary as some ACTs show treatment failure in the GMS. For example, a patient with a *Plasmodium falciparum* infection returning from Southeast Asia should not be treated with dihydroartemisinin–piperaquine. Instead, patients returning from this region could be treated with artesunate–mefloquine or artemether–lumefantrine.

### Reflection

A limitation of this study is the small number of countries included. However, most of the countries included face at least 50 cases of imported malaria annually. Because there is, to our knowledge, no overview on malaria prevalence outside endemic regions, other non‐endemic countries with substantial numbers of imported malaria may have been missed in this review. Furthermore, we have no insight into which extent clinicians follow the national guidelines or the WHO guidelines. This review assessed guidelines solely focussing on the treatment of *Plasmodium falciparum* malaria. A similar review on the treatment of other malaria species will be conducted in the near future.

## CONCLUSION

In conclusion, many national malaria treatment guidelines are not in line with WHO recommendations. Too often, non‐ACTs such as atovaquone–proguanil and quinine are recommended as first‐line treatment. When an ACT is advised as the first choice, few guidelines follow‐up with an alternative ACT as the second choice. An ACT should not only be the first but also the second choice of treatment, in children, adults and second and third trimester pregnant women. Furthermore, most guidelines do not mention artemisinin and partner drug resistance, especially in the GMS. Failing to distinguish between travellers from the GMS and other parts of the world could lead to therapy failure in the first group. We recommend keeping a travellers' origin in mind, especially in the case of treatment failure and choosing a suitable ACT based on regional resistance patterns.
